# Noise control for molecular computing

**DOI:** 10.1098/rsif.2018.0199

**Published:** 2018-07-11

**Authors:** Tomislav Plesa, Konstantinos C. Zygalakis, David F. Anderson, Radek Erban

**Affiliations:** 1 Mathematical Institute, University of Oxford, Andrew Wiles Building, Radcliffe Observatory Quarter, Woodstock Road, Oxford, UK; 2 School of Mathematics, University of Edinburgh, Maxwell Building, Peter Guthrie Tait Road, Edinburgh, UK; 3 Department of Mathematics, University of Wisconsin–Madison, Lincoln Drive, Madison, WI, USA

**Keywords:** synthetic biology, biochemical engineering, molecular/DNA computing, chemical reaction networks, stochastic dynamical systems, intrinsic noise

## Abstract

Synthetic biology is a growing interdisciplinary field, with far-reaching applications, which aims to design biochemical systems that behave in a desired manner. With the advancement in nucleic-acid-based technology in general, and strand-displacement DNA computing in particular, a large class of abstract biochemical networks may be physically realized using nucleic acids. Methods for systematic design of the abstract systems with prescribed behaviours have been predominantly developed at the (less-detailed) deterministic level. However, stochastic effects, neglected at the deterministic level, are increasingly found to play an important role in biochemistry. In such circumstances, methods for controlling the intrinsic noise in the system are necessary for a successful network design at the (more-detailed) stochastic level. To bridge the gap, the *noise-control algorithm* for designing biochemical networks is developed in this paper. The algorithm structurally modifies any given reaction network under mass-action kinetics, in such a way that (i) controllable state-dependent noise is introduced into the stochastic dynamics, while (ii) the deterministic dynamics are preserved. The capabilities of the algorithm are demonstrated on a production–decay reaction system, and on an exotic system displaying bistability. For the production–decay system, it is shown that the algorithm may be used to redesign the network to achieve noise-induced multistability. For the exotic system, the algorithm is used to redesign the network to control the stochastic switching, and achieve noise-induced oscillations.

## 1. Introduction

Synthetic biology is an interdisciplinary field of science and engineering that aims to construct biochemical systems with prescribed behaviours [1,2]. At the theoretical level, the synthetic systems may significantly enhance our understanding of biology. At the practical level, they may have broad applications, e.g. in medicine [3–7], industry [8,9] and nanotechnology [10,11]. The systems may also be of interest to space agencies for optimizing extraterrestrial explorations [12]. A proof of concept for synthetic biology is a synthetic oscillator called the repressilator, which was implemented *in vivo* [13]. The experimental advances since the repressilator range from isolated synthetic biochemical networks, to microorganisms containing partially, or even fully, synthetic DNA molecules (synthetic life) [14–17]. Examples include microorganisms containing a synthetic bistable switch [18], and a cell-density controlling quorum sensor [19], microorganisms producing antimalarial drugs [6,7], and synthetic systems designed for tumour detection, diagnosis and adaptive drug-response [4,5].

The construction of biochemical networks in synthetic biology may be broken down into two steps: firstly, an abstract system is constructed, displaying prescribed properties, and taking the form of a chemical reaction network [20–22]. Secondly, the abstract network is mapped to a suitable physical network, which may then be integrated into a desired environment (e.g. a test-tube, a vesicle or a living cell) [23–26]. Let us note that the first step generally consists of a number of sub-steps, involving mathematical analyses and computational verifications, depending on the nature of the target physical network [23,27] (see also §2.3 and electronic supplementary material).

In the first step of network construction, the goal is to obtain an abstract network with desired dynamics. In this paper, we consider reaction networks under mass-action kinetics: it is assumed that each reaction fires at the rate proportional to the product of the concentrations of the underlying reacting species. In this setting, we consider two dynamical models of reaction networks [22,28]: the deterministic model and the stochastic model (see electronic supplementary material for more details). The deterministic model takes the form of the reaction-rate equations, which are ordinary differential equations governing the time-evolution of the species concentrations [22,28]. The stochastic model takes the form of a Markov chain, which may be simulated using the Gillespie stochastic simulation algorithm [29]. The Gillespie algorithm generates random copy-number time-series, with the copy-number distribution matching that obtained from the underlying chemical master equation [22,28–30]. The stochastic model is more detailed, taking into an account the discreteness of the species counts and the stochastic nature of the dynamics, which may be particularly important in biochemistry, where reaction networks may contain low-abundance species [13,18,21,31–35]. On the other hand, the deterministic model is less detailed, and more appropriate when the species are in high abundance, and the discreteness and stochasticity are negligible [36].

In the second step of network construction, the goal is to engineer a physical network whose dynamics match well the dynamics of a given abstract network, over a suitable time interval. Engineering an appropriate physical network may proceed indirectly, by altering a preexisting physical network, or directly, by engineering a network, which involves a given set of physical species, from scratch. The advantage of the former approach is that a preexisting network may display (partially) desirable dynamical properties. However, such a network may involve DNA and RNA molecules, proteins and metabolites [2], some of which may have complex biophysical properties. Consequently, the disadvantage is that the structure (and, thus, the dynamics) of such a network cannot generally be modified in an arbitrary manner. In the latter approach, one may choose the physical species, at the expense of having to build a network from scratch. This approach is followed in the subfield of nucleic-acid-based molecular computing. For example, in DNA computing, physical networks are engineered with chemical species consisting exclusively of DNA molecules, interacting via the toehold-mediated DNA strand-displacement mechanism [23]. DNA production is systematic and cost-effective, and due to the fact that DNA biophysics is relatively well understood, one has more freedom in controlling the structure of corresponding physical networks. More precisely, an abstract network under mass-action kinetics may be mapped to a DNA-based physical network provided it consists of up to second-order reactions, with rate coefficients varying over up to six orders of magnitude. The resulting physical network has identical deterministic dynamics as the abstract network (in the asymptotic limit of some of the kinetic parameters [23]), up to a scaling of the dependent variables. A proof of concept for DNA computing is a synthetic oscillator called the displacillator, which was implemented *in vitro* [37]. Let us note that DNA-based networks may also be augmented with enzymes [26,38]. Another emerging approach within nucleic-acid-based molecular computing is based on RNA strand displacement [39]—a mechanism which is hypothesized to occur naturally within living cells [40].

The DNA-based reaction networks may involve only high-abundance species, mixed in a test-tube [23]. In such circumstances, it may be sufficient to construct the networks via the (less-detailed) deterministic model. However, recent experimental advancements, involving compartmentalized circuits [24–26], localized circuits [41,42] and molecular robots [43,44], may require reaction network construction via the (more-detailed) stochastic model. For example, in [24–26], the chemical mixture from a test-tube is split into a large number of cell-size vesicles (allowing for an experimental investigation of biochemistry in cell-like reactors). This corresponds to replacing a given reaction network, involving only high-abundance species, with a large number of topologically equivalent networks which, however, may involve species in a low abundance, making the (intrinsic) noise an important part of the dynamics. The intrinsic noise may be controlled in two ways. It may be decreased (e.g. [32]), in order to reduce the differences between the stochastic and deterministic dynamics. On the other hand, it may be increased, in a state-dependent manner, in order to favourably change the stochastic dynamics. In the language of molecular computing, the latter approach corresponds to exploiting the proven computational power of the stochastic reaction networks [45], by reprogramming the underlying intrinsic noise. Let us note that exploitations of the noise for enhancing biological functions have been reported in applications [31,35]. In this paper, we follow the latter approach, and present the noise-control algorithm (given as algorithm 1) which maps an input reaction network to output networks whose stochastic dynamics have an additional controllable state-dependent noise. Importantly, the input and output networks have an identical deterministic model in appropriate limits of some of the parameters introduced by the algorithm. The algorithm may play a significant role in the biochemical network synthesis, allowing for a deterministic–stochastic hybrid approach. More precisely, when constructing abstract and physical networks, one may use the deterministic model to guide the construction [20,21], and then apply the algorithm to favourably modify the intrinsic noise in the stochastic model, while preserving the desired deterministic dynamics. The algorithm may also be used to adjust the intrinsic noise to favourably interact with environment-induced effects (e.g. extrinsic noise).

The rest of the paper is organized as follows. In §2, we introduce algorithm 1 by applying it to the test network (2.1), which at the deterministic level displays a globally attracting equilibrium point. We show that the algorithm can favourably modify the stationary probability distribution underlying (2.1) at arbitrary points of the state-space, without influencing the deterministic dynamics. For example, it is shown that the algorithm may be used to redesign (2.1) to achieve noise-induced multimodality (multistability). In §3, we apply algorithm 1 to the exotic network (3.1), which at the deterministic level displays a bistability involving an equilibrium point and a limit cycle. The algorithm is used to redesign (3.1) to increase the stochastic switching between the two attractors, and to achieve noise-induced oscillations. Finally, in §4, we conclude with a summary and discussion. The notation used in the paper is introduced as needed and is summarized at the beginning of the electronic supplementary material.

## 2. A one-species regular system

Consider the one-species production–decay reaction network 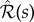, given by (2.1):2.1
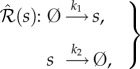
with the reaction-rate equations2.2
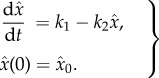
Species *s* from network (2.1) reacts according to the two reactions with rate coefficients 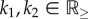, where 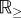 is the set of non-negative real numbers, and 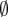 is the zero-species (denoting species which are not of interest). In this paper, we assume reaction networks are under mass-action kinetics, with the reactions taking place in unit-volume reactors. Let us denote the concentration of species *s* from (2.1) at time 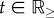 by 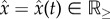. The initial value problem for the deterministic model (also called the drift) for network (2.1) is given by system (2.2), with 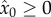 (see also the electronic supplementary material). As the deterministic model (2.2) has a globally attracting equilibrium point, given by *k*_1_/*k*_2_, network (2.1) is said to be regular [22].

**Algorithm 1.** The noise-control algorithm.

**Input:** Let the input reaction network be given by 1.1 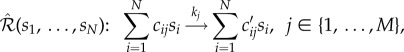 where *s*_1_, …, *s*_*N*_ are the species, *k*_*j*_ the reaction rate coefficients, and *c*_*ij*_, *c*_*ij*_′ the stoichiometric coefficients. **(1) Step:** Reaction network 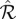 , given by (1.1), is mapped to a *pairwise conservative network* 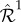 given by 1.2 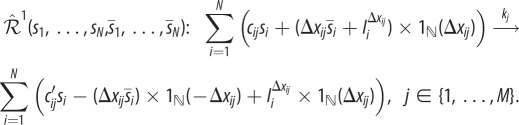 Here, 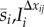 are additional species, Δ*x*_*ij*_ = (*c*_*ij*_′ − *c*_*ij*_), and 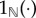 is the indicator function of the natural numbers. **(2) Step:** For each species *I*_*i*_^Δ*x*_*ij*_^, a *drift-corrector network* is constructed, 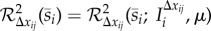 , given by 1.3 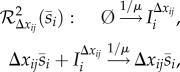 where 0≤*μ*≪1. **(3) Step:** For each species 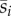 , a union of *zero-drift networks* may be constructed. Let 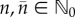 , and 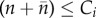 . Network 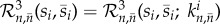 , with 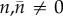 , is given by 1.4 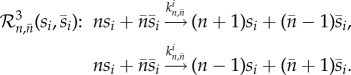 Network 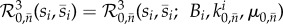 , with 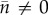 , is given by 1.5 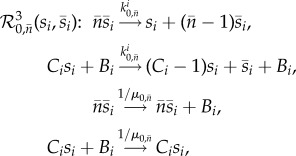 where 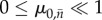 and *B*_*i*_ is an additional species. Network 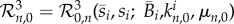 . **Output:** An output reaction network 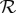 is given by 1.6 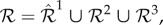 where 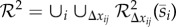 , and 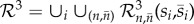 .

Let us denote the copy-number of species *s* from (2.1) at time *t* ≥ 0 by 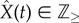, where 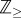 is the set of non-negative integers. Under the stochastic model, 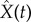 is modelled as a continuous-time discrete-space Markov chain (see also the electronic supplementary material), whose realizations can be generated by using the Gillespie stochastic simulation algorithm [29]. Given 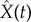, there will be a mean interevent time until one of the reactions from (2.1) fires. The mean interevent time is given by 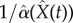, and when the event takes place, the probability that the *i*th reaction from (2.1) fires is 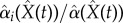, for *i* ∈ {1, 2}. Here, 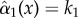, and 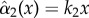, are the so-called propensity functions of the first, and second, reactions from (2.1), respectively. The function 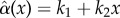 is the total propensity function of network (2.1), i.e. the sum of propensity functions of all the underlying reactions.

We now wish to structurally modify network (2.1) in such a way that (i) the deterministic model from (2.2) is preserved, while (ii) a control is gained over the interevent time from the stochastic model. We accomplish this by, firstly, imposing a conservation law on the target species *s* from network (2.1), thereby truncating its state-space, 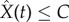, where 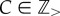 is a conservation constant. The conservation law is imposed in such a way that the total propensity function of the resulting network, denoted by 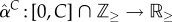, is given by 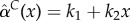, i.e. it has the same form as the total propensity function of the original network (2.1), but is restricted to the bounded discrete domain 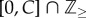. With the restriction imposed, we furthermore embed appropriate reactions to the conservative network, so that an arbitrary non-negative function, denoted by 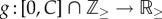, is added to 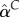, i.e. the resulting total propensity function is given by 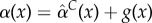. This implies that the interevent time is controllably decreased for any desired state *x*, i.e. in a state-dependent manner. Equivalently, the two requirements imply that a controllable state-dependent noise is introduced into the stochastic dynamics. We have designed a three-step algorithm, given as algorithm 1, which achieves such goals for arbitrary reaction networks under mass-action kinetics. Let us describe properties of the algorithm by applying it on network (2.1).

Firstly, in order to bound the domain of species *s*, an additional species 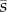 is introduced into network (2.1), in such a way that *s* and 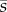 satisfy a pairwise stoichiometric conservation law, formally written 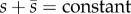. Secondly, to ensure the obtained enlarged network has the same deterministic model as the initial network (2.1), despite the added species 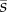, an auxiliary species *I*^1^ is introduced. More precisely, applying the first two steps of the algorithm leads to network 
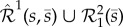
 given by2.3
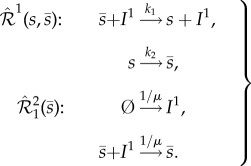
Species 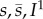 from (2.3) react according to the four reactions with rate coefficients 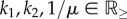. Reaction network 
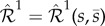
, given in (2.3), is obtained from network 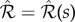, given by (2.1), in the following way: since the first reaction in 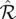 *increases* the copy-number of *s* by one, 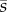 and *I*^1^ are added to the reactants of the reaction, and *I*^1^ is added to the products, leading to the first reaction in 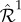. Since the second reaction in 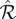 *decreases* the copy-number of *s* by one, 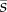 is added to the products, leading to the second reaction in 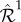. This ensures that the desired conservation law, 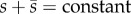, holds. The superscript in *I*^1^ indicates that species *I*^1^ is involved as a catalyst in a reaction of 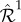 in which *s* is *increased by one*. The subscript in 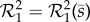 indicates that the network describes production and decay of *I*^1^.

The initial value problem for the deterministic model of (2.3) is given by2.4
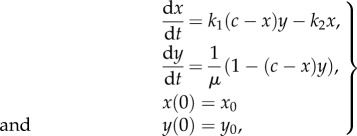
where 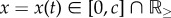, and 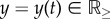, are the concentrations of species *s*, and *I*^1^, from (2.3), respectively, with 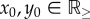 and 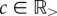. We have used the kinetic conservation law 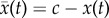, where 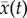 is the concentration of species 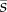, and *c* is a finite time-independent conservation constant. Note that the conservation law truncates the state space of *x*. Let us now describe relationships between systems (2.2) and (2.4), starting with the weak statement: for *c* > *k*_1_/*k*_2_, and for any *μ* > 0, solutions of (2.2) and (2.4) are the same in the long-time limit *t* → ∞. More precisely, the *x*-component of the equilibrium point of (2.4) is identical to the equilibrium point of (2.2), and both are stable. In the electronic supplementary material, we justify the strong statement: for sufficiently large *c*, and for 0 < *μ*≪1, solutions of (2.2) and (2.4), with the same initial conditions, are approximately the same at each time *t* ≥ 0. For these reasons, we call 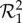 a *drift-corrector network*. Let us note that we have assumed the rate coefficients appearing in subnetwork 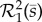 from (2.3) are identical for simplicity, and that this assumption may be relaxed. More precisely, if the rate coefficients of the first and second reactions in 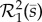 are 1/*μ*_1_ and 1/*μ*_2_, respectively, then the same conclusion from this paragraph holds, provided the rate coefficient *k*_1_ from subnetwork 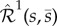 is replaced by (*μ*_1_/*μ*_2_)*k*_1_.

### Zero-drift network 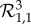


2.1.

Having completed the first two steps, let us focus on the third (and final) step, in which we introduce arbitrary noise into the stochastic model of (2.3), without influencing the deterministic model (2.4). Let us start our consideration by embedding into (2.3) network 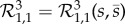, which is given by2.5
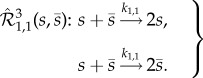
The subscript in 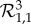 indicates that the underlying reactions have one molecule of *s*, and one of 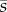, as reactants. The two reactions in (2.5) preserve the conservation law from (2.3). Furthermore, the first and second reactions produce, and degrade, exactly one molecule of *s*, respectively, and they fire at the same rate. Consequently, embedding 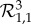 into (2.3) does not affect the underlying deterministic model (2.4), and we call 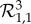 a *zero-drift network*. Note that the deterministic dynamics are not preserved if the rate coefficients in (2.5) are different. However, 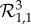 does affect the underlying stochastic model [22,46–48]. To illustrate this, let us consider network 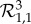 in isolation: the reactions from (2.5) fire when *X*(*t*) ∈ (0, *C*), but not when *X*(*t*) ∈ {0, *C*}, so that 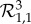 in isolation fires until *X*(*t*) takes one of the extreme values {0, *C*}. Here, 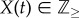, and 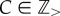, are the copy-number of species *s* appearing in (2.3) and (2.5) at time *t* ≥ 0, and the finite conservation constant, respectively. Note that a possible biologically relevant realization of network (2.5), aside from, e.g. DNA strand-displacement mechanism, is a dimer version of the bifunctional histidine kinase/phosphatase reported in [49].

**Figure 1.**
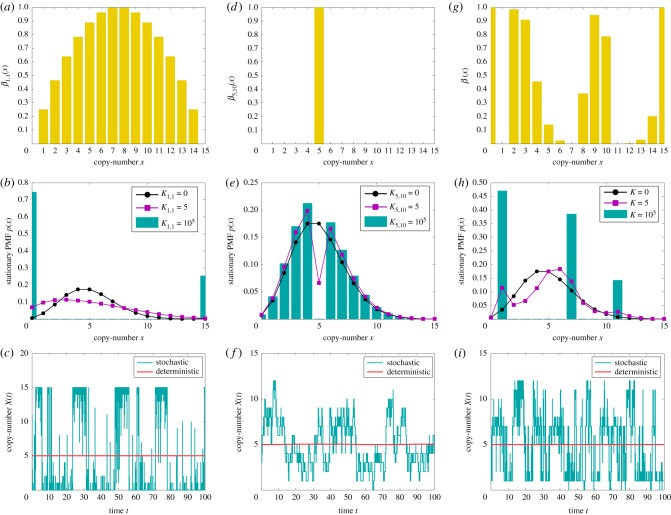
Panels (*a*), (*d*) and (*g*) display propensity functions *β*_1,1_(*x*), *β*_5,10_(*x*) and *β*(*x*)≡*β*_0,15_(*x*) + *β*_2,9_(*x*) + *β*_8,5_(*x*)+*β*_12,0_(*x*), respectively. Panels (*b*), (*e*) and (*h*) display the stationary PMF of networks 
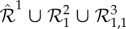
, 
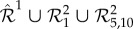
 and 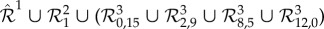, respectively, where 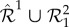 is given by (2.3), while the rest of the (zero-drift) networks are as given in second step of algorithm 1. In (*h*), *K*≡*K*_0,15_ = *K*_2,9_ = *K*_8,5_ = *K*_12,0_. Panels (*c*), (*f*) and (*i*) display in blue the sample paths, corresponding to the PMFs shown as the blue histograms in (*b*), (*e*) and (*h*), respectively, and were obtained by applying the Gillespie algorithm on the underlying networks. Also shown in red are the deterministic trajectories, obtained by numerically solving the corresponding deterministic models. The dimensionless parameters are fixed to: *k*_1_ = 2.5, *k*_2_ = 0.5, *μ* = 10^−3^, *C* = 15, and the state-space for species *I*^1^ is bounded in (*b*), (*e*) and (*h*) by 50. In (*b*) and (*e*), the two-species stationary chemical master equation (CME) was numerically solved, while in (*h*) the boundary zero-drift networks are taken in the asymptotic limits *μ*_0,15_, *μ*_12,0_ → 0. The blue and red trajectories from panel (*i*) were generated with (*μ*_0,15_)^−1^*M*_0,15_ = (*μ*_12,0_)^−1^*M*_12,0_ = 10^7^. The trajectories from (*c*), (*f*) and (*i*) were all initiated at the deterministic equilibrium, *X*(0) = 5.

In the electronic supplementary material, we derive equation (S11) which describes the effective behaviour of the Markov chain *X*(*t*) from network 
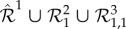
 in the limit *μ* → 0, and it follows that the effective total propensity function of the network, denoted *α*(*x*), satisfies2.6
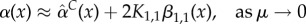
where2.7
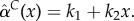
Function 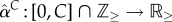 has the form of the total propensity of network (2.1), and *K*_1,1_*β*_1,1_(*x*) is the propensity function of reactions in (2.5), with the scaled factors given by2.8
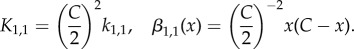
Function *β*_1,1_(*x*) is displayed in figure 1*a*, where one can notice its parabolic shape, arising from the underlying conservation law 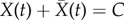, which holds for all *t* ≥ 0, where 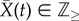 is the copy-number of 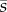 at time *t* ≥ 0. Comparing (2.6) and (2.7), it follows that, as *μ* → 0, the mean interevent time for network 
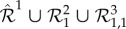
 is lower than for network (2.1), in the regions of the common state space where *β*_1,1_(*x*)≠0, i.e. for *x* ∈ (0, *C*). Coefficient *K*_1,1_ controls by how much the interevent time is reduced. Equivalently, *β*_1,1_(*x*), and *K*_1,1_, determine the support, and magnitude, respectively, of the state-dependent intrinsic noise which network (2.5) introduces into the dynamics of network (2.3).

In the electronic supplementary material, we rigorously formulate the following two approximate results (given as equations (S13) and (S17), respectively):2.9
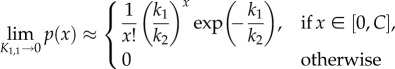
and2.10
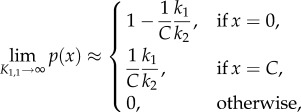
where *p*(*x*) is the stationary probability mass function (PMF) corresponding to network 
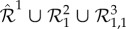
 in the limit *μ* → 0, i.e. the probability that there are *x* molecules of species *s* as *μ* → 0 in the long-time limit *t* → ∞. Let us interpret analytical results (2.9) and (2.10), and compare them with the numerically obtained counterparts. In figure 1*b*, we display numerically obtained stationary *x*-marginal PMFs for different values of *K*_1,1_, with the rest of the (dimensionless) parameters fixed to *k*_1_ = 2.5, *k*_2_ = 0.5, *μ* = 10^−3^ and *C* = 15. It can be seen that, for *K*_1,1_ = 0, i.e. when the zero-drift network 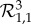 does not fire, the PMF matches that of network (2.1), i.e. it is a Poissonian, as predicted by (2.9). Let us note that the matching of the PMFs of networks (2.1) and 
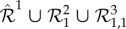
 relies on choosing sufficiently large rate coefficients 1/*μ* in the drift-corrector network 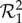. When *K*_1,1_ = 5, the PMF appears closer to a uniform distribution than does the PMF when *K*_1,1_ = 0. Finally, for the larger value *K*_1,1_ = 10^5^, i.e. when zero-drift network 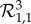 fires much faster than network 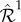, the PMF redistributes across the domain, accumulating at the boundary, and becoming bimodal. This is in qualitative agreement with (2.6), and in quantitative agreement with (2.10), which predicts *p*(0) ≈ 0.7 and *p*(15) ≈ 0.3. In figure 1*c*, a representative sample path is shown, obtained by applying the Gillespie algorithm on network 
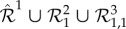
, when *K*_1,1_ = 10^5^. Also shown is a trajectory obtained by numerically solving the deterministic model (2.4). Consistent with figure 1*b*, the sample path switches between the boundary of the state space, with a bias towards the left boundary point *x* = 0. This is in contrast to the deterministic trajectories, which are globally attracted to the equilibrium point *x* = 5.

### General zero-drift networks 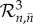


2.2.

The zero-drift network 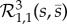, given by (2.5), involves a single molecule of *s* and 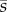 as reactants, and adds the noise at *x* ∈ [1, *C* − 1], i.e. in the interior of the state space. Similar networks may be used to add the noise at any point in the state space, without influencing the deterministic dynamics. In particular, in (1.4) and (1.5), we present general zero-drift networks 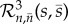, which involve *n* molecules of *s*, and 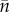 of 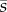, as reactants, and add the noise at 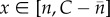, where 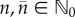, and 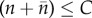. Embedding a union of such networks, 
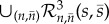
, into (2.3), we arrive at the result similar to (2.6), with *K*_1,1_*β*_1,1_(*x*) replaced by the linear combination 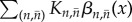. The scaled rate coefficient 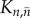 and function 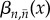 are given in the electronic supplementary material as equations (S18) and (S19), respectively, where we also justify that an arbitrary non-negative function, defined on a bounded discrete domain, may be represented by a suitable sum 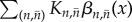.

To illustrate general zero-drift networks, let us start with embedding into network (2.3), with the conservation constant *C* = 15, the zero-drift network 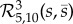, satisfying (1.4) with *n* = 5 and 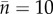. In figure 1*d*, we show propensity function *β*_5,10_(*x*), which is non-zero only at *x* = 5. In figure 1*e*, we show the numerically approximated stationary *x*-marginal PMFs underlying network 
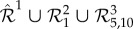
 for different values of *K*_5,10_, with the rest of the parameters as in figure 1*b*. One can notice that, under the action of network 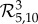, the PMF is gradually decreased to nearly zero at *x* = 5 (the deterministic equilibrium), and becomes bimodal, with the two noise-induced maxima at *x* = 4 and *x* = 6. In figure 1*f*, we show a corresponding representative sample path.

In general, noise-induced multimodality may be achieved by a suitable combination of zero-drift networks. For example, let us synthetize noise such that the stationary PMF is trimodal, and nearly zero everywhere, except at *x* ∈ {1, 7, 11}. Such a task may always be achieved by a suitable combination of the basis zero-drift networks, i.e. those zero-networks that induce noise only at a single point in the state space (e.g. subnetwork 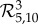 with propensity function shown in figure 1*d*; see also the electronic supplementary material). In the present case, one could construct the 13 basis zero-drift networks which add large enough noise at *x* ∈ [0, 15]\{1, 7, 11}. Here, for simplicity, we achieve the task with only four zero-drift networks. In figure 1*g*–*i*, we consider network 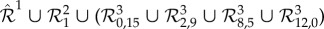. We denote *β*(*x*)≡*β*_0,15_(*x*) + *β*_2,9_(*x*) + *β*_8,5_(*x*) + *β*_12,0_(*x*), and, for simplicity, take *K*≡*K*_0,15_ = *K*_2,9_ = *K*_8,5_ = *K*_12,0_. The resultant propensity function *β*(*x*) is shown in figure 1*g*, while in figure 1*h* it can be seen that the PMF becomes trimodal for sufficiently large *K*, with the maxima at *x* = {1, 7, 11}. This is consistent with the corresponding representative sample path shown in blue in figure 1*i*, which display tristability. Let us note that, while the stochastic dynamics display multistability in figure 1*c,f,i*, the corresponding deterministic dynamics, also shown in the plots, remain monostable.

### 2.3. Compilation into DNA-based networks

Chemical reaction networks, whose stochastic dynamics are controlled by algorithm 1, may be mapped to the nucleic-acid-based ones. The mapping takes a different form depending on which molecular compiler is used and, in this section, we briefly outline two approaches. Firstly, the molecular compiler put forward in [23], based on 4-domain signal strands, requires that the input reaction network consists of up to second-order reactions. On the other hand, let us note that it allows reactions with identical reactants (as is the case in zero-drift networks). Thus, one is generally required to apply a single pre-compiling step, where the higher-order reactions (i.e. reactions involving three or more reactants) are approximated by systems of up to second-order ones [50,51], before using the 4-domain DNA compiler. However, the 4-domain compiler has only been shown to preserve the deterministic dynamics when mapping an abstract network into a DNA-based one [23]. In the electronic supplementary material, we show that the stochastic dynamics are also preserved, making the compiler compatible with the noise-control algorithm. Furthermore, we apply the compiler to a network of the form (2.3) and (2.5), and briefly discuss the pre-compilation step, leaving the details for a future publication [51]. On the other hand, the 2-domain molecular compiler put forward in [27], and experimentally implemented in [52], can be used directly, without any pre-compilation, since it automatically handles higher-order reactions.

## 3. A two-species exotic system

**Figure 2.**
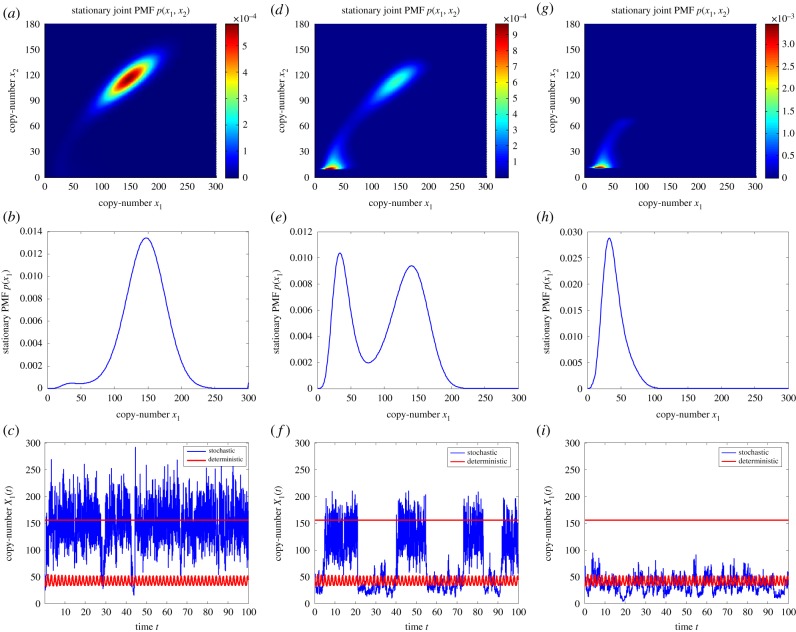
Panel (*a*) displays the joint stationary PMF of network (3.1), while (*d*) and (*g*) display the stationary PMFs of network (S35) from electronic supplementary material, for (*K*_0,*C*_2_ − 10_, *K*_30,0_) = (10^18^, 2 × 10^8^) and (*K*_0,*C*_2_ − 10_, *K*_30,0_) = (10^18^, 10^18^), respectively, with the rest of the parameters being the same. Panels (*b*), (*e*) and (*h*) display the *x*_1_-marginal PMFs corresponding to (*a*), (*b*) and (*c*), respectively. Panels (*c*), (*f*) and (*i*) display in blue the sample paths, corresponding to the PMFs shown in (*b*), (*e*) and (*h*), respectively, and were obtained by applying the Gillespie algorithm on the underlying networks. Also shown in red are two deterministic trajectories, one initiated near the equilibrium point, while the other near the limit cycle, obtained by numerically solving equation (S34) from electronic supplementary material. The dimensionless parameters are fixed to: *k*_1_ = 4, *k*_2_ = 1.408, *k*_3_ = 0.0518, *k*_4_ = 0.164, *k*_5_ = 3.1 × 10^−3^, *k*_6_ = 4.8 × 10^−3^, *k*_7_ = 4, *k*_8_ = 8, *k*_9_ = 0.16, *k*_10_ = 0.104, *k*_11_ = 2.1 × 10^−3^. In (*a*)–(*b*), (*d*)–(*e*) and (*g*)–(*h*), the stationary chemical master equation (CME) is numerically solved, with the state-space truncated to (*x*_1_, *x*_2_) ∈ [0, *C*_1_] × [0, *C*_2_], where *C*_1_ = 300, *C*_2_ = 180 and *μ*, *μ*_0,*C*_2_ − 10_, *μ*_30,0_ → 0. The blue sample paths from panels (*f*) and (*i*) were generated with (*μ*^−1^, (*μ*_0,*C*_2_ − 10_)^−1^*M*_0,*C*_2_ − 10_, (*μ*_30,0_)^−1^*M*_30,0_) = (10^3^, 10^20^, 2 × 10^10^) and (*μ*^−1^, (*μ*_0,*C*_2_ − 10_)^−1^*M*_0,*C*_2_ − 10_, (*μ*_30,0_)^−1^*M*_30,0_) = (10^3^, 10^20^, 10^20^), respectively. The blue trajectories from (*c*), (*f*) and (*i*) were all initiated near the deterministic limit cycle.

Consider the two-species network 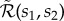 given by3.1
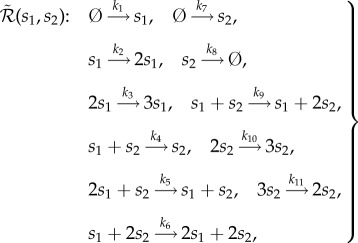
where species *s*_1_ and *s*_2_ react according to the 11 reactions with rate coefficients *k*_1_, *k*_2_, …, *k*_11_≥0. We denote the copy-numbers of species *s*_1_, and *s*_2_, at time *t* by *X*_1_(*t*), and *X*_2_(*t*), respectively. It was established in [20] that, for particular choices of the rate coefficients, the deterministic model of reaction network (3.1), given as equation (S34) in the electronic supplementary material, exhibits exotic dynamics: it undergoes a homoclinic bifurcation, and displays a bistability involving a limit cycle and an equilibrium point. On the other hand, it is demonstrated in [21] that the stochastic model of (3.1) is not necessarily sensitive to the deterministic bifurcation, and may effectively behave in a monostable manner. The latter point is demonstrated in figure 2*c*, where we show in red numerically approximated *x*_1_-solutions of equation (S34) from the electronic supplementary material, one initiated in the region of attraction of the equilibrium point, while the other of the limit cycle. For a comparison, we also show in blue a representative sample path generated by applying the Gillespie algorithm on (3.1). It can be seen that the stochastic solution spends significantly more time near the deterministic equilibrium point. To gain a clearer picture, we display in figure 2*a*,*b* the joint, and the *x*_1_-marginal, stationary PMFs, respectively, underlying network (3.1), which have been obtained numerically for the same parameter values as in figure 2*c*. In figure 2*b*, one can notice that the PMF is bimodal, but the left peak, corresponding to the limit cycle, is significantly smaller than the right peak, which corresponds to the stable equilibrium point.

We now apply algorithm 1 on network (3.1) to achieve two goals. Firstly, we balance the sizes of the two peaks of the stationary PMF from figure 2*b*, thereby forcing the stochastic system to spend comparable amounts of time at the two deterministic attractors. Secondly, we reverse the situation shown in figure 2*b*, by making the left PMF peak significantly larger than the right one, thereby forcing the stochastic system to spend most of the time near the limit cycle. We could achieve the goals by introducing species 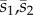 into (3.1), and using suitable basis zero-drift networks. We take a simpler approach, by mapping (3.1) to 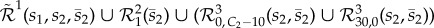, which is given as equation (S35) in the electronic supplementary material. For our purposes, only one of 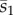, 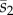 is sufficient, as the stochastic dynamics of *s*_1_ and *s*_2_ are coupled. We have chosen 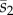 for convenience, since *x*_2_-state-space may be truncated at a lower value, *C*_2_ = 180, than *x*_1_-state-space (see also figure 2*a*). The *x*_2_-component of the deterministic limit cycle satisfies *x*_2_ ∈ (10, 30). Correspondingly, we introduce two zero-drift networks: 
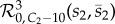
, and 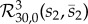, which redistribute the PMF from *x*_2_ ∈ [0, 10], and from *x*_2_ ∈ [30, *C*_2_], respectively, to the limit cycle region, *x*_2_ ∈ (10, 30). We fix the scaled rate coefficient *K*^2^_0,*C*_2_ − 10_ to a large value (so that the PMF is nearly zero for *x*_2_ ∈ [0, 10]), and vary the coefficient *K*^2^_30,0_, which redistributes the PMF from the deterministic equilibrium point to the limit cycle. Network 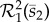 is necessary for the preservation of the deterministic dynamics of (3.1) under the application of algorithm 1.

In figure 2*d*,*e*, we show the joint, and *x*_1_-marginal, stationary PMFs for an intermediate value of *K*^2^_30,0_, when the PMF is partially redistributed from *x*_2_ ∈ [30, *C*_2_] to *x*_2_ ∈ (10, 30), so that the two peaks in figure 2*e* are of comparable sizes. In figure 2*f*, we show a representative sample path, obtained by applying the Gillespie algorithm on network (S35), together with the deterministic trajectories obtained by solving (S34). One can notice that the stochastic system now spends significantly more time near the limit cycle, when compared to figure 2*c*. Let us note that stochastic switching between a coexisting equilibrium point and a limit cycle in a DNA-based reaction network has been observed experimentally [26]. In figure 2*f*,*g*, we show analogous plots, but for a sufficiently large value of *K*^2^_30,0_, when the PMF is almost completely redistributed from *x*_2_ ∈ [30, *C*_2_] to *x*_2_ ∈ (10, 30). Now, in contrast to figure 2*a*–*c*, the PMF becomes unimodal, and concentrated around the limit cycle. Let us note that the red trajectories from figure 2*f*,*i* were generated by numerically solving the deterministic model (S34) from the electronic supplementary material. For our purposes, it is not necessary to solve the corresponding (stiff) deterministic model of network (S35) from the electronic supplementary material. The reason is that algorithm 1 does not influence the deterministic equilibrium points of a given reaction network, regardless of the choice of the kinetic algorithm parameters. Thus, while the deterministic limit cycle is not necessarily preserved for the algorithm parameters chosen in figure 2*i*, the enclosed deterministic unstable focus is necessarily preserved. Consequently, the blue sample path shown corresponds to noise-induced oscillations either near a deterministic limit cycle, or near a deterministic unstable focus.

## 4. Discussion

In this paper, we have presented the noise-control algorithm, which is given as algorithm 1. The algorithm maps an input chemical reaction network to output networks, all under mass-action kinetics, by introducing appropriate additional species and reactions, such that the output networks satisfy the following two properties. Firstly, the output networks have the same deterministic model as the input network, in appropriate limits of some of the parameters (rate coefficients) introduced by the algorithm. Secondly, controllable state-dependent noise is introduced into the stochastic model of the output networks. Thus, algorithm 1 may be used to control the intrinsic noise of a given reaction network under mass-action kinetics, while preserving the deterministic dynamics. Let us note that the asymptotic conditions for the algorithm parameters are necessary for preservation of the time-dependent deterministic solutions. However, the time-independent deterministic solutions (the deterministic equilibrium points), which capture important features of the deterministic dynamics, are preserved under the algorithm even if the asymptotic conditions are not satisfied.

The algorithm has been applied to a test problem, taking the form of the one-species production–decay system given by (2.1). Using analytical and numerical methods, we have shown that the additional intrinsic noise, introduced by the algorithm, may be used to favourably modify the stationary PMF at arbitrary points in the state space, as demonstrated in figure 1. For example, in figure 1*b*, the noise is added to the whole interior of the state space, while in (*e*) only at a single point, in both cases resulting in noise-induced bimodality. On the other hand, in figure 1*h*, by adding the noise to specific points in the state space, the network is redesigned to display noise-induced trimodality. As shown in figure 1*c*,*f*,*i*, the blue stochastic trajectories display multistability, while the red deterministic ones remain monostable.

The algorithm has also been applied to a more challenging problem, taking the form of the two-species system given by (3.1), which, for the parameters taken in this paper, at the deterministic level displays a bistability involving an equilibrium point and a limit cycle [20,21]. At the stochastic level, the system is significantly more likely to be found near the equilibrium point, as demonstrated in figure 2*a*–*c*. We have used the algorithm to redesign network (3.1), so that the stochastic system spends comparable amounts of time near the two attractors, as demonstrated in figure 2*d*–*f*. The network was also redesigned to display noise-induced oscillations, which is shown in figure 2*g*–*i*. Put another way, one may view the dynamics shown in figure 2*a*–*c* as being contaminated by the noise, which disrupts the oscillatory chemical computation. Algorithm 1 has been applied to address the disruption by appropriately reprogramming the noise. Such control is of practical relevance, since stochastic switching between an equilibrium and a limit cycle has already been observed experimentally in a DNA-based network [26].

The controllable state-dependent noise is generated by algorithm 1 using the zero-drift networks (1.4) and (1.5). Any non-negative function, defined on a bounded discrete domain, may be represented by a linear combination of propensity functions induced by an appropriate union of the zero-drift networks. Thus, choosing suitable zero-drift networks, the algorithm may control the intrinsic noise at arbitrary points in the state space of the stochastic dynamics of reaction networks. The cost of such a precision in noise control is a larger number of reactants in the underlying zero-drift networks. However, while the high-molecular reactions introduced by the algorithm are more expensive to synthetize, they do not limit applicability of algorithm 1 to DNA computing. The reason for this is that such reactions may always be broken down into sets of up to bimolecular reactions, with asymptotically equivalent dynamics [50,51], as outlined in §2.3 and exemplified in the supplementary material. Let us stress that the lower the molecular copy-numbers of a given reaction network are, the more important it becomes to control their stochastic behaviour, and, fortunately, the *less* costly algorithm 1 becomes (since the zero-drift networks involve fewer reactants).

Algorithm 1 constitutes a qualitatively novel scientific discovery which will facilitate the progress of nucleic-acid-based computing, such as DNA computing [23–26]. In particular, we put forward a hybrid approach for constructing DNA-based reaction networks: the deterministic model may be used to guide the construction of reaction networks, and then algorithm 1 may be applied to favourably reprogram the intrinsic noise in the stochastic model, while preserving the mean-field behaviour. Put another way, the deterministic–stochastic hybrid approach allows one to reshape the probability distributions of target chemical species, while inheriting the fixed mean-field behaviour. This provides a control over, not only the probability distributions of the chemical species, but also over their sample paths, as e.g. demonstrated in §3 with a noisy limit cycle. Furthermore, the algorithm does not depend on the initial conditions of the underlying species, beyond the conservation laws. This is in contrast to the methods presented in [53,54], which do not attempt to control the sample-path behaviour, and whose performance depends strongly on the initial conditions of the underlying species, which may impose significant experimental challenges. The noise-control algorithm may be of critical importance when the synthetic networks involve species at low copy-numbers, since then the stochastic effects may play a significant role [13,18,21,24–26,31–35]. On the one hand, the algorithm may enhance our understanding of biology, via theoretical and experimental investigations of the role of intrinsic noise in both prebiotic and biotic chemical processes [24–26]. On the other hand, the algorithm may facilitate *in vivo* implementations of synthetic DNA networks, where the reactions may take place at a cellular level. In such circumstances, without a control, the noise may contaminate the performance of the synthetic networks. Algorithm 1 provides a way to control the stochastic effects, enriching the DNA-based synthetic systems with novel, noise-induced functionalities. For example, one may envisage using the algorithm to design nucleic-acid-based circuits interacting favourably with gene-regulatory networks, where noise-induced multimodality is known to play a critical role [55,56]. On the one hand, the algorithm could be used to induce multimodality in the probability distribution of an appropriate intracellular protein, resulting in cell phenotype diversity. On the other hand, the algorithm could also be used to make the protein distribution narrower around the single peak, thus inducing a cell phenotype robustness.

## Supplementary Material

Additional Examples and Analysis

## Data accessibility

Data supporting this paper are contained within the paper, and as the electronic supplementary material.
