# Supplementary material for: Noise control for molecular computing
Source: J R Soc Interface. 2018 Jul 11;15(144):20180199. doi: 10.1098/rsif.2018.0199 (PMC6073653; doi:10.1098/rsif.2018.0199)
Supplement: Additional Examples and Analysis [file rsif20180199supp1.pdf]

Supplementary material for Noise control for molecular computing

Plesa et al., Journal of the Royal Society Interface

Notation. Set $\mathbb{R}$ is the space of real numbers, $\mathbb{R}_{\geq}$ the space of nonnegative real numbers, and $\mathbb{R}_{>}$ the space of positive real numbers. Similarly, $\mathbb{Z}$ is the space of integer numbers, $\mathbb{Z}_{\geq}$ the space of nonnegative integer numbers, and $\mathbb{Z}_{>}$ the space of positive integer numbers. Given two real numbers $a, b \in \mathbb{R}$, open interval $\{x \in \mathbb{R} | a < x < b\}$ is denoted by (a,b), while closed interval $\{x \in \mathbb{R} | a \leq x \leq b\}$ by $[a,b]$. Given sets $\mathcal{R}_1$ and $\mathcal{R}_2$, their union is denoted by $\mathcal{R}_1 \cup \mathcal{R}_2$, while their intersection by $\mathcal{R}_1 \cap \mathcal{R}_2$. *Support* of function $f : \mathbb{Z} \to \mathbb{R}$ is defined by $\text{supp}(f) = \{x \in \mathbb{Z} | f(x) \neq 0\}$.

1. Dynamical models of chemical reaction networks

Let us consider the mass-action reaction network $\mathcal{R}$ given by

$$\mathcal{R}(s_1,\ldots,s_N): \sum_{i=1}^{N} c_{ij} s_i \xrightarrow{k_j} \sum_{i=1}^{N} c'_{ij} s_i, \quad j \in \{1,\ldots,M\}, \tag{S1}$$

where $s_1,\ldots,s_N$ are the reacting species, k_j the reaction rate coefficients, and c_{ij}, c'_{ij} the stoichiometric coefficients. Let us denote by $\mathbf{c}_j, \mathbf{c}'_j \in \mathbb{N}_0^N$ the vectors of the stoichiometric coefficients of reaction j, and let $\Delta \mathbf{x}_j = \mathbf{c}'_j - \mathbf{c}_j$.

The *deterministic model* of reaction network (S1) is given by the following system of ordinary-differential equations (ODEs), known as the reaction-rate equations [1]:

$$\frac{\mathrm{d}\mathbf{x}}{\mathrm{d}t} = \sum_{j=1}^{M} k_j \mathbf{x}^{\mathbf{c}_j} \Delta \mathbf{x}_j, \quad i \in \{1,\ldots,N\}. \tag{S2}$$

Here, $\mathbf{x} = \mathbf{x}(t) \in \mathbb{R}_{\geq}^N$ is the vector of species concentrations, i.e. $x_i(t)$ is the concentration of species s_i at time t, and $\mathbf{x}^{\mathbf{c}_j} = \prod_{l=1}^N x_l^{c_{lj}}$, with the convention $0^0 = 1$.

The *stochastic model* of reaction network (S1) is given by the following system of partial difference-differential equations, known as the chemical master equation (CME) [1, 2]:

$$\frac{\partial}{\partial t} p(\mathbf{x},t) = \mathcal{L} p(\mathbf{x},t) = \sum_j (E_{\mathbf{x}}^{-\Delta \mathbf{x}_j} - 1)\big(\alpha_j(\mathbf{x}) p(\mathbf{x},t)\big). \tag{S3}$$

Here, $p(\mathbf{x},t)$ is the probability mass function (PMF), i.e. the probability that the vector of copy-numbers $\mathbf{X} = \mathbf{X}(t) \in \mathbb{Z}_{\geq}^N$ of species $s_1,\ldots,s_N$ at time t is given by $\mathbf{x}$. Linear operator $\mathcal{L}$ is called the forward operator, and step operator $E_{\mathbf{x}}^{-\Delta \mathbf{x}_j}$ is such that $E_{\mathbf{x}}^{-\Delta \mathbf{x}_j} p(\mathbf{x},t) = p(\mathbf{x} - \Delta \mathbf{x}_j, t)$. Function $\alpha_j(\mathbf{x})$ is the propensity function [1] of the j-th reaction from (S1), and is given by

$$\alpha_j(\mathbf{x}) = k_j \mathbf{x}^{\underline{\mathbf{c}_j}} = k_j \prod_{l=1}^{N} x_l^{\underline{c_{lj}}}, \tag{S4}$$

where $x_l^{\underline{c_{lj}}}$ denotes a falling factorial of x_l, i.e. $x_l^{\underline{c_{lj}}} = x_l(x_l - 1)\ldots(x_l - c_{lj} + 1)$, with the convention $x^{\underline{0}} = 1$ for all $x \in \mathbb{Z}_{\geq}$.

2. Analysis of dynamical models of network $\hat{\mathcal{R}}^1 \cup \mathcal{R}_1^2 \cup \mathcal{R}_{1,1}^3$

In what follows, we analyse the deterministic and stochastic models of the output reaction network $\hat{\mathcal{R}}^1 \cup \mathcal{R}_1^2 \cup \mathcal{R}_{1,1}^3$, consisting of the subnetworks given by (3) and (5) in the paper, in the asymptotic limit $\mu \to 0$ (when the drift-corrector network $\mathcal{R}_1^2$ fires infinitely fast). We also analyse the stochastic model in the asymptotic limits $K_{1,1} \to 0$, and $K_{1,1} \to \infty$ (when the zero-drift network $\mathcal{R}_{1,1}^3$ does not fire, and when it fires infinitely fast, respectively). The obtained limiting dynamics of the output network are compared with the dynamics of the input network $\hat{\mathcal{R}}$, given by (1) in the paper.

2.1 The deterministic model in the limit $\mu \to 0$

Let us analyse equation (4), from the paper, in the asymptotic limit $\mu \to 0$. It follows from the Tikhonov theorem [3] that the ODE for y, given by second equation in (4), reduces to the algebraic equation $y = (c-x)^{-1}$ as $\mu \to 0$. Substituting the algebraic equation into (4) results in

$$\begin{aligned} \frac{\mathrm{d}x}{\mathrm{d}t} &= k_1 - k_2 x, \\ x(0) &= x_0, \text{ as } \mu \to 0. \end{aligned} \tag{S5}$$

Initial value problems (2), from the paper, and (S5) have the same form, and let us denote their solutions by $\hat{x}(t; \hat{x}_0)$ and $x(t; x_0)$, respectively. Then, choosing the conservation constant $c \geq \max_{t \geq 0} \hat{x}(t; \hat{x}_0) < \infty$, and $x_0 = \hat{x}_0$, ensures that the concentration of auxiliary species $\bar{s}$ is nonnegative, $\bar{x}(t) = c - x(t) \geq 0$, and that the solutions of (2) and (4) are asymptotically equivalent in the limit $\mu \to 0$.

2.2 The stochastic model in the limit $\mu \to 0$

CME induced by network $\hat{\mathcal{R}}^1 \cup \mathcal{R}_1^2 \cup \mathcal{R}_{1,1}^3$ is given by

$$\frac{\partial}{\partial t} p(x,y,t) = \left(\mathcal{L}^1 + \frac{1}{\mu} \mathcal{L}_1^2 + K_{1,1} \mathcal{L}_{1,1}^3 \right) p(x,y,t), \tag{S6}$$

where $x(t), y(t) \in \mathbb{Z}_{\geq}$ are copy-numbers of species s, I^1 from (3), respectively, with

$$\begin{aligned} \mathcal{L}^1 &= k_1 (E_x^{-1} - 1)\left((C - x) y\right) + k_2 (E_x^{+1} - 1) x, \\ \mathcal{L}_1^2 &= (E_y^{-1} - 1) + (C - x)(E_y^{+1} - 1) y, \\ \mathcal{L}_{1,1}^3 &= (E_x^{-1} + E_x^{+1} - 2) \beta_{1,1}(x), \end{aligned} \tag{S7}$$

and $K_{1,1}, \beta_{1,1}(x)$ given in equation (8), in the paper. Operators $\mathcal{L}^1, \mathcal{L}_1^2, \mathcal{L}_{1,1}^3$ are induced by subnetworks $\hat{\mathcal{R}}^1, \mathcal{R}_1^2, \mathcal{R}_{1,1}^3$, respectively.

Let us analyse system (S6) in the limit $\mu \to 0$, and consider the following perturbation series:

$$p(x,y,t) = p_0(x,y,t) + \mu p_1(x,y,t) + \dots + \mu^i p_i(x,y,t) + \dots, \tag{S8}$$

with $i \geq 2$. Substituting (S8) into (S6), equating terms of equal powers in μ, and writing $p_0 = p_0(x,y,t)$, $p_1 = p_1(x,y,t)$, the following system of equations is obtained:

$$\begin{aligned} \mathscr{O}\left(\frac{1}{\mu}\right) &: \ \mathscr{L}_1^2 p_0 = 0, \\ \mathscr{O}(1) &: \ -\mathscr{L}_1^2 p_1 = (\mathscr{L}^1 + K_{1,1}\mathscr{L}_{1,1}^3 - \frac{\partial}{\partial t})p_0. \end{aligned} \tag{S9}$$

Order $1/\mu$ equation. Let us write $p_0(x,y,t) = p_0(y|x)p_0(x,t)$, where $p_0(y|x)$ is the stationary PMF of y conditional on x, while $p_0(x,t)$ is the marginal PMF of x. Substituting $p_0(x,y,t) = p_0(y|x)p_0(x,t)$ into the first equation in (S9), with t and x fixed, leads to $-\mathscr{L}_1^2 p_0(y|x) = 0$. It follows that $p_0(y|x)$ is the Poisson distribution with parameter $(C-x)^{-1}$, so that the zero-order PMF is given by

$$p_0(x,y,t) = \left(\frac{1}{y!}\left(\frac{1}{(C-x)}\right)^y \exp\left(-\frac{1}{(C-x)}\right)\right) p_0(x,t). \tag{S10}$$

Order 1 equation. Substituting (S10) into the second equation in (S9), summing over all the possible states $y \in \mathbb{Z}_\geq$, using (S7), and equalities $\sum_y y p_0(y|x) = (C-x)^{-1}$ and $\sum_y p_0(y|x) = 1$, one obtains the *effective CME*, given by

$$\frac{\partial}{\partial t} p_0(x,t) = \left(\mathscr{L} + K_{1,1}\mathscr{L}_{1,1}^3\right) p_0(x,t), \tag{S11}$$

where $\mathscr{L}$ is the forward operator corresponding to network (1), and has the following form

$$\mathscr{L} = k_1(E_x^{-1} - 1) + k_2(E_x^{+1} - 1)x. \tag{S12}$$

2.2.1 Limit $K_{1,1} \to 0$

Setting the left-hand side to zero, and taking $K_{1,1} = 0$, in (S11), and assuming C is fixed to a sufficiently large value, it follows that the stationary PMF is a Poisson distribution with parameter k_1/k_2 [2]:

$$p_0(x) = \begin{cases} \frac{1}{x!}\left(\frac{k_1}{k_2}\right)^x \exp\left(-\frac{k_1}{k_2}\right), & \text{if } x \in [0,C], \\ 0, & \text{otherwise.} \end{cases} \tag{S13}$$

2.2.2 Limit $K_{1,1} \to \infty$

Let us substitute the perturbation series

$$p_0(x) = f_0(x) + \frac{1}{K_{1,1}} f_1(x) + \dots + \left(\frac{1}{K_{1,1}}\right)^i f_i(x) + \dots, \tag{S14}$$

with $i \geq 2$, into (S11) with the left-hand side set to zero, and consider the limit $K_{1,1} \to \infty$. Then, equating terms of equal powers in $1/K_{1,1}$, one obtains:

$$\begin{aligned} \mathscr{O}(K_{1,1}) &: \ \mathscr{L}_{1,1}^3 f_0(x) = 0, \\ \mathscr{O}(1) &: \ -\mathscr{L}_{1,1}^3 f_1(x) = \mathscr{L} f_0(x). \end{aligned} \tag{S15}$$

Order $K_{1,1}$ equation. The solution to the first equation in (S15) is given by

$$f_0(x) = \begin{cases} 1 - \frac{a}{C}, & \text{if } x = 0, \\ \frac{a}{C}, & \text{if } x = C, \\ 0, & \text{otherwise,} \end{cases} \tag{S16}$$

where $a \in \mathbb{R}_\geq$ is an arbitrary constant.

Order 1 equation. Multiplying the second equation in (S15) by x, and summing over $x \in \mathbb{Z}_\geq$, with the convention that $f_0(x) = 0$ and $\beta_{1,1}(x) = 0$ for $x \notin [0,C]$, one obtains the solvability condition $0 = \sum_{x=0}^{\infty} x \mathscr{L} f_0(x)$, which implies $a = k_1/k_2$. Substituting a into (S16) leads to the zero-order approximation of the stationary PMF:

$$f_0(x) = \begin{cases} 1 - \frac{1}{C}\frac{k_1}{k_2}, & \text{if } x = 0, \\ \frac{1}{C}\frac{k_1}{k_2}, & \text{if } x = C, \\ 0, & \text{otherwise.} \end{cases} \tag{S17}$$

3. Zero-drift networks $\mathscr{R}_{n,\bar{n}}^3$

The propensity function of reactions underlying $\mathscr{R}_{n,\bar{n}}^3(s,\bar{s})$, $n,\bar{n} \in \mathbb{Z}_\geq$, and $(n+\bar{n}) \leq C$, is given by $K_{n,\bar{n}}\beta_{n,\bar{n}} : [0,C] \to \mathbb{R}_\geq$, with

$$K_{n,\bar{n}} = M_{n,\bar{n}} k_{n,\bar{n}}, \tag{S18}$$

and

$$\beta_{n,\bar{n}}(x) = (M_{n,\bar{n}})^{-1} x^{\underline{n}} (C-x)^{\underline{\bar{n}}}, \tag{S19}$$

where the scaling factor $M_{n,\bar{n}}$ is introduced to approximately normalize $\beta_{n,\bar{n}}(x)$, and is given by

$$M_{n,\bar{n}} = \left(\frac{n}{n+\bar{n}}C\right)^{\underline{n}} \left(\frac{\bar{n}}{n+\bar{n}}C\right)^{\underline{\bar{n}}}. \tag{S20}$$

Function $\beta_{n,\bar{n}}(x)$ is nonzero on the interval $[n, C-\bar{n}]$, with the unique maximum approximately at $Cn/(n+\bar{n})$.

Interior zero-drift networks. Zero-drift network $\mathscr{R}_{n,\bar{n}}^3(s,\bar{s})$, with $n,\bar{n} \neq 0$, satisfies equation (15) in the paper, and the propensity function of its reactions, which is proportional to (S19), is nonzero only in the interior of the state-space. Since the propensity function of $\mathscr{R}_{n,\bar{n}}^3(s,\bar{s})$, with $n,\bar{n} \neq 0$, attains its maximum in the interior of the domain, we call the network an interior zero-drift network.

Boundary zero-drift networks. Network $\mathscr{R}_{0,\bar{n}}^3(s,\bar{s})$, satisfying equation (16) in the paper, is a zero-drift network in the limit $\mu_{0,\bar{n}} \to 0$. Furthermore, in the same limit, the

first two reactions from (16) have the same propensity function, which is proportional to (S19) with $n = 0$, and which is nonzero at the left boundary point, $x = 0$. Similarly, network $\mathscr{R}^3_{n,0} = \mathscr{R}^3_{0,n}(\bar{s}, s; \bar{B}, k_{n,0}, \mu_{n,0})$ is a zero-drift network as $\mu_{n,0} \to 0$, and its first two reactions have the same propensity function, which is nonzero at the right boundary point, $x = C$. Since networks with $n = 0$ (respectively, $\bar{n} = 0$) generate propensity functions with the maximum values at the left (respectively, right) boundary point, we call such networks left (respectively, right) boundary zero-drift networks.

Basis zero-drift networks. Stoichiometric coefficients $n, \bar{n}$ control the support of the intrinsic noise, which network $\mathscr{R}^3_{n,\bar{n}}$ introduces into the stochastic dynamics, via the control of support of function (S19). The larger the sum $(n + \bar{n})$ is, with $(n + \bar{n}) \le C$, the smaller the support of (S19), and hence one obtains a more precise noise-control. In the special case when $n + \bar{n} = C$, the propensity function (S19) is nonzero only at a single point in the state-space, $x = n$. We call networks $\mathscr{R}^3_{n,\bar{n}}(s, \bar{s})$, with $n + \bar{n} = C$, basis zero-drift networks, and the corresponding propensity functions *basis propensity functions*. Any nonnegative function, defined on a bounded discrete domain, may be represented by a suitable linear combination of the basis propensity functions.

4. Stochastic DNA Compiler

In this section, we analyze the 4-domain DNA compiler from [4], which has been shown to preserve the deterministic dynamics when mapping suitable abstract networks to the DNA-based ones. We show that the compiler also preserves the stochastic dynamics. Furthermore, we apply the compiler to a reaction network designed using the noise-control algorithm, and briefly outline the pre-compiling step: how to approximate higher-order reactions with up-to second order ones. Let us note that we change the notation slightly in this section, in order to match it with the notation from [4].

First-order reactions. Consider an arbitrary input first-order reaction

$$X_m \xrightarrow{k} \sum_{i=1}^{N} c_i X_i, \tag{S21}$$

involving abstract species $X_1, X_2, \ldots, X_N$, and the following output DNA-based second-order network

$$\begin{aligned} X_m + G &\xrightarrow{\varepsilon k} O, \\ O + T &\xrightarrow{q} \sum_{i=1}^{N} c_i X_i, \end{aligned} \tag{S22}$$

where $X_1, X_2, \ldots, X_N$ represent target single-stranded DNA molecules, O is an intermediate single-stranded DNA, and G and T are double-stranded DNAs called a gate and a translator, respectively [4]. Here, we assume rate coefficients k and q are of order one, $k, q = \mathscr{O}(1)$, with respect to the asymptotic parameter $0 < \varepsilon \ll 1$. Furthermore, we assume that the copy-numbers of the reservoir species G and T are initially given by $G(0) = T(0) = 1/\varepsilon \equiv N_{\max} \in \mathbb{Z}_>$, where we abuse the notation slightly by denoting identically the species and their corresponding copy-numbers. It has been shown that reaction (S21) and network (S22) are approximately deterministically identical over suitable time-intervals for sufficiently small ε. We now show that this is also true at the stochastic level.

To this end, let us firstly assume ε is chosen small enough, so that species G and T remain approximately fixed over a desired time-interval $[0, \Omega]$, $\Omega \ge 0$, i.e. $G(t) \approx 1/\varepsilon$ and $T(t) \approx 1/\varepsilon$ for $t \in [0, \Omega]$, and so that network (S22) may then be approximated by

$$\begin{aligned} X_m &\xrightarrow{k} O, \\ O &\xrightarrow{q/\varepsilon} \sum_{i=1}^{N} c_i X_i. \end{aligned} \tag{S23}$$

Denoting the copy-numbers of species X_i and O by x_i and o, respectively, and defining new coordinates $\bar{x}_i = (x_i + c_i o)$ for $i \in \{1, 2, \ldots, N\}$, the CME induced by (S23) reads as

$$\frac{\partial}{\partial t} p(\bar{\mathbf{x}}, o, t) = \left(\frac{1}{\varepsilon} \mathscr{L}_0 + \mathscr{L}_1 \right) p(\bar{\mathbf{x}}, o, t), \tag{S24}$$

where

$$\begin{aligned} \mathscr{L}_0 &= (E_o^{+1} - 1) q o, \\ \mathscr{L}_1 &= (E_{\bar{\mathbf{x}}}^{-\Delta \bar{\mathbf{x}}} E_o^{-1} - 1) k (\bar{x}_m - c_m o), \end{aligned}$$

and with $\Delta \bar{x}_i = c_i$ for $i \neq m$, and $\Delta \bar{x}_m = c_m - 1$. Substituting the perturbation series $p(\bar{\mathbf{x}}, o, t) = p_0(\bar{\mathbf{x}}, o, t) + \varepsilon p_1(\bar{\mathbf{x}}, o, t) + \ldots$ into (S24), one obtains the hierarchy of equations given by

$$\begin{aligned} \mathscr{O}\left(\frac{1}{\varepsilon}\right) &: \ \mathscr{L}_0 p_0(\bar{\mathbf{x}}, o, t) = 0, \\ \mathscr{O}(1) &: \ -\mathscr{L}_0 p_1(\bar{\mathbf{x}}, o, t) = (\mathscr{L}_1 - \frac{\partial}{\partial t}) p_0(\bar{\mathbf{x}}, o, t). \end{aligned} \tag{S25}$$

The solution to the first equation from (S25) is given by $p_0(\bar{\mathbf{x}}, o, t) = p_0(\bar{\mathbf{x}}, t)\delta_{o,0}$, where $\delta_{i,j}$ is the Kronecker-delta function ($\delta_{j,j} = 1$, and $\delta_{i,j} = 0$ for $i \neq j$). Thus, it follows that o converges to zero in probability, implying that $\bar{\mathbf{x}} \to \mathbf{x}$. Summing the second equation from (S25) over variable o, one finally obtains the effective CME: $\frac{\partial}{\partial t} p_0(\mathbf{x}, t) = (E_{\mathbf{x}}^{-\Delta \mathbf{x}} - 1) k x_m$. Since this matches the CME of the input reaction (S21), we have established that (S21) and (S22) match stochastically in a weak sense.

Second-order reactions. Consider now an arbitrary input second-order reaction

$$X_m + X_n \xrightarrow{k} \sum_{i=1}^{N} c_i X_i, \tag{S26}$$

and the following output DNA-based second-order network

$$X_m + L \underset{q}{\overset{k}{\rightleftharpoons}} H + B,$$
$$X_n + H \xrightarrow{q} O,$$
$$O + T \xrightarrow{q} \sum_{i=1}^{N} c_i X_i, \qquad \text{(S27)}$$

where $X_1, X_2, \ldots, X_N$ represent target single-stranded DNA molecules, B and O are auxiliary single-stranded DNAs, while L, H and T are auxiliary double-stranded DNAs [4]. Here, $\rightleftharpoons$ denotes a reversible reaction (which is equivalent to two irreversible reactions). We assume that the copy-numbers of the reservoir species L, B and T are initially given by $L(0) = B(0) = T(0) = 1/\varepsilon = N_{\max} \in \mathbb{Z}_>$. Furthermore, for simplicity, let us also assume that the rate coefficient q is chosen to be significantly larger than k, i.e. $k = \mu q$, with $0 < \mu \ll 1$. Such an assumption may be relaxed by appropriately rescaling the rate coefficients appearing in (S27) [4]. We now show that reaction (S26) and network (S27) are approximately dynamically identical over suitable time-intervals at the stochastic level for sufficiently small ε, and $\mu/\varepsilon \equiv \lambda = \mathcal{O}(1)$.

Assume ε is chosen small enough, so that $L(t) \approx 1/\varepsilon$, $B(t) \approx 1/\varepsilon$, $T(t) \approx 1/\varepsilon$ over the desired time-interval $t \in [0, \Omega]$, and network (S27) is then approximately given by

$$X_m \underset{q/\varepsilon}{\overset{\lambda q}{\rightleftharpoons}} H,$$
$$X_n + H \xrightarrow{q} O,$$
$$O \xrightarrow{q/\varepsilon} \sum_{i=1}^{N} c_i X_i. \qquad \text{(S28)}$$

Let us denote the copy-numbers of species X_i, H and O by x_i, h and o, respectively, and assume that species X_m and X_n are distinct, i.e. that $m \neq n$. Defining new coordinates $\bar{x}_i = x_i + c_i o$ for $i \neq m$, and $\bar{x}_m = x_m + c_m o + h$, and a slow time-scale $\tau = \mathcal{O}(1)$ by $\tau = \varepsilon t$, and substituting into the resulting CME the perturbation series $p(\bar{\mathbf{x}}, h, o, \tau) = p_0(\bar{\mathbf{x}}, h, o, \tau) + \varepsilon p_1(\bar{\mathbf{x}}, h, o, \tau) + \varepsilon^2 p_2(\bar{\mathbf{x}}, h, o, \tau) + \ldots$, one obtains the following system of equations

$$\mathcal{O}\left(\frac{1}{\varepsilon^2}\right): \ \mathcal{L}_0 p_0(\bar{\mathbf{x}}, h, o, \tau) = 0,$$
$$\mathcal{O}\left(\frac{1}{\varepsilon}\right): \ -\mathcal{L}_0 p_1(\bar{\mathbf{x}}, h, o, \tau) = \mathcal{L}_1 p_0(\bar{\mathbf{x}}, h, o, \tau),$$
$$\mathcal{O}(1): \ -\mathcal{L}_0 p_2(\bar{\mathbf{x}}, h, o, \tau) = \mathcal{L}_1 p_1(\bar{\mathbf{x}}, h, o, \tau) - \frac{\partial}{\partial \tau} p_0(\bar{\mathbf{x}}, h, o, \tau). \qquad \text{(S29)}$$

Here, operators $\mathcal{L}_0$ and $\mathcal{L}_1$ are given by

$$\mathcal{L}_0 = (E_h^{+1} - 1) q h + (E_o^{+1} - 1) q o,$$
$$\mathcal{L}_1 = (E_h^{-1} - 1) \lambda q (\bar{x}_m - c_m o - h) + (E_{\bar{\mathbf{x}}}^{-\Delta \bar{\mathbf{x}}} E_h^{+1} E_o^{-1} - 1) q (\bar{x}_n - c_n o) h,$$

where $\Delta \bar{x}_i = c_i$ for $i \notin \{n, m\}$ and $\Delta \bar{x}_i = c_i - 1$ for $i \in \{n, m\}$.

The solution to the first equation from (S29) is given by $p_0(\bar{\mathbf{x}}, h, o, \tau) = p_0(\bar{\mathbf{x}}, \tau) \delta_{h,0} \delta_{o,0}$. A solution to the second equation is given by $p_1(\bar{\mathbf{x}}, h, o, \tau) = p_0(\bar{\mathbf{x}}, \tau) \delta_{o,0} p_1(h)$, where the third factor satisfies $h p_1(h) = \lambda E_h^{-1} (\bar{x}_m - h) \delta_{h,0}$. Let us note that o and h approach zero as $\varepsilon \to 0$, implying that $\bar{\mathbf{x}} \to \mathbf{x}$. Finally, substituting the obtained solutions into the third equation from (S29), and summing over variables h and o, one obtains the effective CME $\frac{\partial}{\partial t} p_0(\mathbf{x}, t) = (E_{\mathbf{x}}^{-\Delta \mathbf{x}} - 1) k x_m x_n p_0(\mathbf{x}, t)$, which is identical to the CME of the input reaction (S26) with $m \neq n$. Applying similar reasoning when $m = n$ also leads to the correct effective CME: $\frac{\partial}{\partial t} p_0(\mathbf{x}, t) = (E_{\mathbf{x}}^{-\Delta \mathbf{x}} - 1) k x_m (x_m - 1) p_0(\mathbf{x}, t)$, with $\Delta x_i = c_i$ for $i \neq m$, and $\Delta x_m = c_m - 2$.

4.1 An Example

Let us now apply the molecular compiler on the reaction network

$$\mathcal{R}^1: \quad \bar{s} \xrightarrow{k_1} s, \qquad s \xrightarrow{k_2} \bar{s},$$
$$\mathcal{R}^3_{1,1}: \quad s + \bar{s} \xrightarrow{k_{1,1}} 2s, \qquad \bar{s} + s \xrightarrow{k_{1,1}} 2\bar{s}. \qquad \text{(S30)}$$

Network (S30) has been obtained by applying Algorithm 1 from the paper on the conservative input network $\bar{s} \xrightarrow{k_1} s$, $s \xrightarrow{k_2} \bar{s}$, so that the second step of the algorithm may be omitted. It is similar to the network consisting of subnetworks given by (3) and (5) in the paper, and is chosen here for simplicity. Replacing s by X_1, and $\bar{s}$ by X_2, to match the notation from [4], a DNA-based implementation of (S30) is given by

$$\bar{\mathcal{R}}^1: \quad X_2 + G_1 \xrightarrow{k_1/N_{\max}} O_1, \quad O_1 + T_1 \xrightarrow{q} X_1, \quad X_1 + G_2 \xrightarrow{k_2/N_{\max}} O_2, \quad O_2 + T_2 \xrightarrow{q} X_2,$$
$$\bar{\mathcal{R}}^3_{1,1}: \quad X_1 + L_3 \underset{q}{\overset{k_{1,1}}{\rightleftharpoons}} H_3 + B_3, \quad X_2 + H_3 \xrightarrow{q} O_3, \quad O_3 + T_3 \xrightarrow{q} 2X_1,$$
$$X_2 + L_4 \underset{q}{\overset{k_{1,1}}{\rightleftharpoons}} H_4 + B_4, \quad X_1 + H_4 \xrightarrow{q} O_4, \quad O_4 + T_4 \xrightarrow{q} 2X_2, \qquad \text{(S31)}$$

where we require that the initial copy-numbers of the auxiliary species G_i, T_i, for $i = 1, \ldots, 4$, and L_i, B_i, for $i = 1, 2$, are all initially set to a sufficiently large value $N_{max} \in \mathbb{Z}_>$, and that q

is set to a sufficiently large value, ensuring $k_{1,1} \ll q$ (note that this condition may be relaxed [4]).

If, instead of the zero-drift network $\mathscr{R}^3_{1,1}$, we embed e.g. the zero-drift network $\mathscr{R}^3_{2,2}$ into network $\mathscr{R}^1$ from (S30), which is given by

$$\begin{aligned}\mathscr{R}^3_{2,2}: \quad & 2s + 2\bar{s} \xrightarrow{k_{2,2}} 3s + \bar{s}, \\ & 2s + 2\bar{s} \xrightarrow{k_{2,2}} s + 3\bar{s}, \qquad \text{(S32)}\end{aligned}$$

then a pre-compilation step is performed. In particular, we first approximate (S32) by a set of up-to bimolecular reactions e.g. as follows [5, 6]:

$$\begin{aligned}\bar{\mathscr{R}}^3_{2,2}: \quad & s + \bar{s} \underset{1/\varepsilon}{\overset{K_1}{\rightleftharpoons}} Q_1, \\ & s + Q_1 \underset{1/\varepsilon}{\overset{K_2}{\rightleftharpoons}} Q_2, \\ & \bar{s} + Q_2 \xrightarrow{K_3} s + Q_2, \\ & \bar{s} + Q_2 \xrightarrow{K_3} 2\bar{s} + Q_1, \qquad \text{(S33)}\end{aligned}$$

where Q_1 and Q_2 are auxiliary species. Provided $\varepsilon^2 K_1 K_2 K_3 = k_{2,2}$, $\varepsilon K_i \ll 1$, for $i = 1,2,3$, and $0 < \varepsilon \ll 1$, networks (S32) and (S33) are approximately dynamically identical. As opposed to the original fourth-order network (S32), the approximating second-order network (S33) can be mapped to a DNA-based one using the compiler from [4].

In this section, we have verified one way to structurally compile an input abstract network, involving low molecular copy-numbers, into a DNA-based output network. Provided the target species copy-numbers are sufficiently low, so that the noise introduced by the zero-drift networks is significant without requiring too large rate coefficients, it may be achievable to constrain all the rate coefficients within six orders of magnitude. Thus, in principle, low copy-number networks designed using the noise-control algorithm are experimentally realizable.

5. Network $\tilde{\mathscr{R}}$

The deterministic model of reaction network $\tilde{\mathscr{R}}$, given by equation (11) in the paper, is given by

$$\begin{aligned}\frac{dx_1}{dt} &= k_1 + k_2 x_1 + k_3 x_1^2 - k_4 x_1 x_2 - k_5 x_1^2 x_2 + k_6 x_1 x_2^2, \\ \frac{dx_2}{dt} &= k_7 - k_8 x_2 + k_9 x_1 x_2 + k_{10} x_2^2 - k_{11} x_2^3, \qquad \text{(S34)}\end{aligned}$$

where $x_1 = x_1(t), x_2 = x_2(t)$ are the concentrations of species s_1, s_2, respectively, at time t.

5.1 An application of the noise-control algorithm

Network $\tilde{\mathscr{R}}^1(s_1,s_2,\bar{s}_2) \cup \mathscr{R}^2_1(\bar{s}_2) \cup (\mathscr{R}^3_{0,C_2-10}(s_2,\bar{s}_2) \cup \mathscr{R}^3_{30,0}(s_2,\bar{s}_2))$, arising from an application of the noise-control algorithm on network $\tilde{\mathscr{R}}$, is given by

$$\begin{aligned}\tilde{\mathscr{R}}^1(s_1,s_2,\bar{s}_2): \quad & \varnothing \xrightarrow{k_1} s_1, \\ & s_1 \xrightarrow{k_2} 2s_1, \\ & 2s_1 \xrightarrow{k_3} 3s_1, \\ & s_1 + s_2 \xrightarrow{k_4} s_2, \\ & 2s_1 + s_2 \xrightarrow{k_5} s_1 + s_2, \\ & s_1 + 2s_2 \xrightarrow{k_6} 2s_1 + 2s_2, \\ & \bar{s}_2 + I_2^1 \xrightarrow{k_7} s_2 + I_2^1, \\ & s_2 \xrightarrow{k_8} \bar{s}_2, \\ & s_1 + s_2 + \bar{s}_2 + I_2^1 \xrightarrow{k_9} s_1 + 2s_2 + I_2^1, \\ & 2s_2 + \bar{s}_2 + I_2^1 \xrightarrow{k_{10}} 3s_2 + I_2^1, \\ & 3s_2 \xrightarrow{k_{11}} 2s_2 + \bar{s}_2, \\ \mathscr{R}^2_1(\bar{s}_2): \quad & \varnothing \xrightarrow{1/\mu} I_2^1, \\ & \bar{s}_2 + I_2^1 \xrightarrow{1/\mu} \bar{s}_2, \\ \mathscr{R}^3_{0,C_2-10}(s_2,\bar{s}_2): \quad & (C_2-10)\bar{s}_2 \xrightarrow{k^2_{0,C_2-10}} s_2 + (C_2-11)\bar{s}_2, \\ & C_2 s_2 + B_2 \xrightarrow{k^2_{0,C_2-10}} (C_2-1)s_2 + \bar{s}_2 + B_2, \\ & (C_2-10)\bar{s}_2 \xrightarrow{1/\mu_{0,C_2-10}} (C_2-10)\bar{s}_2 + B_2, \\ & C_2 s_2 + B_2 \xrightarrow{1/\mu_{0,C_2-10}} C_2 s_2, \\ \mathscr{R}^3_{30,0}(s_2,\bar{s}_2): \quad & 30s_2 \xrightarrow{k^2_{30,0}} 29s_2 + \bar{s}_2, \\ & C_2\bar{s}_2 + \bar{B}_2 \xrightarrow{k^2_{30,0}} s_2 + (C_2-1)\bar{s}_2 + \bar{B}_2, \\ & 30s_2 \xrightarrow{1/\mu_{30,0}} 30s_2 + \bar{B}_2, \\ & C_2\bar{s}_2 + \bar{B}_2 \xrightarrow{1/\mu_{30,0}} C_2\bar{s}_2. \qquad \text{(S35)}\end{aligned}$$

References

[1] Anderson DF, Kurtz TG. Stochastic analysis of biochemical systems. Springer; 2015.

[2] Van Kampen NG. Stochastic processes in physics and chemistry. Elsevier; 2007.

[3] Klonowski W. Simplifying principles for chemical and enzyme reaction kinetics. Biophysical Chemistry, 1983;18(3):73–87.

[4] Soloveichik D, Seeling G, Winfree E. DNA as a universal substrate for chemical kinetics. Proceedings of the National Academy of Sciences, 2010;107(12):5393–5398.

[5] Wilhelm T. Chemical systems consisting only of elementary steps - a paradigma for nonlinear behavior. Journal of Mathematical Chemistry, 2000;27:71–88.

[6] Plesa T. Stochastic approximation of high-molecular by bi-molecular reactions. In preparation; 2018.
